# Effect of alternating bioremediation and electrokinetics on the remediation of *n*-hexadecane-contaminated soil

**DOI:** 10.1038/srep23833

**Published:** 2016-04-01

**Authors:** Sa Wang, Shuhai Guo, Fengmei Li, Xuelian Yang, Fei Teng, Jianing Wang

**Affiliations:** 1 Institute of Applied Ecology, Chinese Academy of Sciences, Shenyang 110016, China; 2 University of Chinese Academy of Sciences, Beijing 100049, China; 3 Shenyang University, Shenyang 110014, China; 4 Institute of Biology, Shandong Academy of Sciences, Jinan 250014, China

## Abstract

This study demonstrated the highly efficient degradation of *n*-hexadecane in soil, realized by alternating bioremediation and electrokinetic technologies. Using an alternating technology instead of simultaneous application prevented competition between the processes that would lower their efficiency. For the consumption of the soil dissolved organic matter (DOM) necessary for bioremediation by electrokinetics, bioremediation was performed first. Because of the utilization and loss of the DOM and water-soluble ions by the microbial and electrokinetic processes, respectively, both of them were supplemented to provide a basic carbon resource, maintain a high electrical conductivity and produce a uniform distribution of ions. The moisture and bacteria were also supplemented. The optimal DOM supplement (20.5 mg·kg^−1^ glucose; 80–90% of the total natural DOM content in the soil) was calculated to avoid competitive effects (between the DOM and *n*-hexadecane) and to prevent nutritional deficiency. The replenishment of the water-soluble ions maintained their content equal to their initial concentrations. The degradation rate of *n*-hexadecane was only 167.0 mg·kg^−1^·d^−1^ (1.9%, w/w) for the first 9 days in the treatments with bioremediation or electrokinetics alone, but this rate was realized throughout the whole process when the two technologies were alternated, with a degradation of 78.5% ± 2.0% for the *n*-hexadecane after 45 days of treatment.

There has been a wealth of research into improving the remediation efficiency of organic-contaminated soils^1^,^2^,^3^,^4^,^5^. Traditional bioremediation and biological intensifying measures that have been widely used include the addition of surfactants^6^, exogenous nutrients^7^ and chemical oxidants^8^,^9^ and the use of electrokinetics^10^. These techniques aim to provide optimal reaction conditions. To enhance microbial remediation by increasing microbial metabolism, dissolved organic matter (DOM) is an important co-substrate and has been recognized as one of the most critical carbon sources^11^. Furthermore, the DOM concentration affects the quantity and degradation abilities of microbes^8^,^12^. Han *et al*.^13^ found that the addition of DOM could enhance the microbial degradation of organic contaminants in soils; similar results were shown in increases in the solubility and biodegradation of contaminants due to the exogenous application of DOM^14^,^15^. However, a competitive effect can be induced if excessive concentrations of DOM are added, which is not conductive to biodegradation^16^,^17^,^18^. Therefore, the optimum range for the DOM content should be determined.

Electrokinetic remediation is a green and powerful remediation technology that has been extensively studied for use in organic-contaminated soils, in terms of the electrochemical mechanisms of electromigration, electroosmosis and electrophoresis^19^. The results have shown improvements in the removal of organic contaminants from soils^20^,^21^. However, electrokinetic remediation changes the soil properties, including the soil pH, moisture and microbial biomass^22^,^23^; these effects limit the current that can be applied^24^, which limits the efficiency of electrokinetic remediation in long-term treatment processes^10^. Polarity reversal has been shown to limit the changes in soil properties^25^,^26^, but if employed, water-soluble ions need to be added to replenish their losses and improve the heterogeneous distribution of the ions in non-uniform electric fields.

The use of electrokinetic techniques in combination with bioremediation vastly improves the efficiency over that of electrokinetic remediation alone in removing organic pollutants from soil^27^,^28^,^29^,^30^. For example, the remediation efficiency was prominently improved using electrokinetic–bioremediation (EK-Bio) in soils contaminated with petroleum and its components^25^,^31^,^32^. The EK-Bio degradation process is highly efficient during the initial stages of treatment owing to the stimulation of bacterial activity by the weak electric field^31^,^32^. However, the remediation rates decrease over time, as the soil DOM and inorganic ion contents change and impact both the bioremediation and electrokinetic processes. Therefore, a combined method that precludes the interference between the bioremediation and electrokinetic technologies should be developed. The purpose of this study was to combine the highly efficient degradation phases of bioremediation and electrokinetics to realize a rapid remediation process for *n*-hexadecane-contaminated soil.

This experiment explored a feasible method in which bioremediation and electrokinetic techniques were alternated to avoid mutual interference. The soil moisture, DOM, and water-soluble ion contents and the microbial community were supplemented to ensure that the soil micro-environment was optimal for the microbial and electrokinetic remediation processes. *n*-Hexadecane, a middle-chain model compound that represents aliphatic hydrocarbons, was used as the test pollutant; it is a chemically stable and persistent pollutant due to its nonpolar properties and has been similarly used in other studies^33^,^34^.

## Materials and Methods

### Soil and chemicals

The soil used in the present study was classified as sandy loam. It was obtained from the Institute of Applied Ecology experimental station, located in Shenyang, China. The soil, taken at 0–10 cm depth, had a slightly alkaline pH and soil organic carbon content typical of the region (Table 1). The collected soil was air-dried and sieved through a 2 mm mesh to create a consistent and homogeneous mix. To artificially contaminate the soil for the experiment, it was mixed with *n*-hexadecane (>98%; Sinopharm Chemical Reagent Company, Shanghai, China). The density of the *n*-hexadecane in the analytical grade reagent was 0.77 g·cm^−3^, and its melting point was 18.3 °C. An *n*-hexadecane-*n*-hexane solution was slowly mixed into the soil, and the soil- *n*-hexadecane-*n*-hexane mixture was agitated in a ventilation hood for two weeks to ensure uniform distribution of the *n*-hexadecane until the *n*-hexane was thoroughly evaporated. NH_4_NO_3_, (NH_4_)_2_SO_4_ and KH_2_PO_4_ (analytical grade, Sinopharm Chemical Reagent Company, China) were also mixed into uncontaminated soil (control treatments; cEK and cBio1) to allow for observations of changes in the soil characteristics. K_2_HPO_4_, MgSO_4_, NaCl, CaCl_2_ and FeSO_4_ (analytical grade, Sinopharm Chemical Reagent Company, China) were prepared for the nutrient solution. Dichloromethane and acetone, used for extracting *n*-hexadecane from the soil, were analytical grade (Sinopharm Chemical Reagent Company, China).

### Bacteria cultures

Two types of petroleum-degrading mixed bacterial culture (Culture A and Culture B) were used, depending on the treatment stage. Culture A was isolated from oil-contaminated soil adjacent to Liaohe Oil Field (Liaoning province, Northeast China), and Culture B was prepared from soil samples removed at the end of the first EK stage (EK1) in the Bio1 + EK1 + Bio2 + EK2 treatment (see ‘Experimental design’). The nutrient solution used for the bacterial cultures was maintained at pH 7.0 and consisted of KH_2_PO_4_ (2.75 g·L^−1^), K_2_HPO_4_ (2.25 g·L^−1^), MgSO_4_ (0.2 g·L^−1^), (NH_4_)_2_SO_4_ (1.0 g·L^−1^), NaCl (0.5 g·L^−1^), CaCl_2_ (0.02 g·L^−1^), FeSO_4_ (0.02 g·L^−1^), a microelement stock solution (1 ml·L^−1^) and petroleum (0.5 g·L^−1^). The bacteria were cultivated on a constant temperature shaker at 30 °C and 180 rpm, and their growth was monitored by measuring the absorption of the culture at OD600 nm; they were harvested by centrifugation during their exponential phase of growth. The bacterial precipitate was resuspended in mineral media to form a bacterial suspension, and only Culture A was evenly mixed into the soil for the initial experiment.

### Experimental design

Seven treatment regimes, summarized in Table 2, were performed in duplicate: controls of each technology type without contamination (cBio1 and cEK), a control with contamination but without bacteria or electrokinetic remediation (CK), a bioremediation-only treatment with Culture A (Bio1), an electrokinetic remediation only treatment (EK), a bio–electrokinetic simultaneous remediation treatment (Bio1-EK), and a treatment in which the applications of bioremediation and electrokinetic remediation were alternated (Bio1 + EK1 + Bio2 + EK2). In the treatments that used contaminated soil, it was contaminated with approximately 1% (v/w) *n*-hexadecane (8.9 g·kg^−1^). The moisture content of the soil in all treatments was maintained at 20% (w/w) by adding deionized water once every 6 days during the electrokinetic treatment stages. For cBio1 and cEK, inorganic ions were added, including NH_4_NO_3_, (NH_4_)_2_SO_4_ and KH_2_PO_4_, to amplify the ionic changes that occurred under each treatment technology. Nutrient solution was added to all of the bioremediation and electrokinetic remediation treatments (Bio1, EK, Bio1-EK, Bio1 + EK1 + Bio2 + EK2; Table 2). At the start of the bioremediation treatments, bacteria Culture A was added to achieve an initial concentration of ca. 4.5 × 10^8^ copies·g^−1^ of dry soil. For the Bio1 + EK1 + Bio2 + EK2 treatment, before the “Bio2” stage, the soil was thoroughly mixed and bacteria Culture B (microbial supplement), nutrient solution, and glucose (as an easily utilizable carbon source) were added to promote microbial metabolism. The added glucose compensated for the loss of the DOC, according to its consumption in the cBio1 and cEK treatments; these treatments were carried out because *n*-hexadecane can interfere with the extraction of the DOM. Thus, by treating uncontaminated soil, the variations in the concentration and distribution of the DOM and inorganic ions in the soil could be monitored. In the Bio1 + EK1 + Bio2 + EK2 treatment, the points of switching between the distinct treatment methods were determined by decreases in the observed degradation rates of *n*-hexadecane. Each experiment was conducted at room temperature (25 ± 1 °C). The completed duration for CK, Bio1, EK and Bio1-EK was 54 days; for the cBio1, cEK and Bio1 + EK1 + Bio2 + EK2 treatments it was 45 days.

### Electrokinetic apparatus

**Figure 1:**
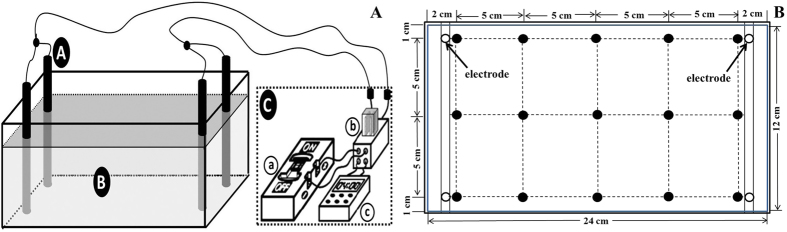
Schematic diagram of the electrokinetic reactor (**A**) and the distribution of the sampling positions (**B**). Within (**A**), ⓐ is the electrode, ⓑ is the contaminated or uncontaminated soil, and ⓒ is the power supply system (including ⓐ, power; ⓑ, relay and ⓒ, timer). Within (**B**), the filled black circles (●) represent the sampling points within the contaminated or uncontaminated soil cells.

The electrokinetic apparatus (Fig. 1A) consisted of a Perspex soil chamber with inner dimensions (L × W × H) of 24 × 12 × 12 cm, a power supply, two pairs of electrodes and an electrode control system. The electrodes were made of graphite, with a length of 15 cm and a diameter of 0.5 cm, and were inserted parallel to each other at either end of the test soil cell. An electric field was generated with a constant voltage gradient of 1.3 V·cm^−1^. The polarity of the electric field was alternated, by the electrode auto-control system, every 2 hours during the electrokinetic treatment processes (Table 2).

### Sample collection

In the electrokinetic and bioremediation treatments (Bio1, EK, Bio1-EK and Bio1 + EK1 + Bio2 + EK2), samples were collected every 3 days to monitor the degradation rate of *n*-hexadecane, and the same was done for CK. A total of 15 samples (5 × 3 samples in each chamber) were collected, and all samples were evenly mixed together before determining the *n*-hexadecane concentrations (Fig. 1B).

For the cBio1 and cEK tests, 15 samples (5 × 3 samples in each chamber) were gathered every 9 days. The three samples from each sampling line (Fig. 1B) were mixed uniformly to measure the water-soluble ions, according to their distance from the electrodes (Fig. 1B). A composite sample of all samples mixed together was also prepared to determine the total ion and dissolved organic matter (DOM) content. All soil samples were stored at −20 °C until analysis.

Finally, the soil electrical conductivity was measured in the EK, EK1 and EK2 samples under a soil to water ratio of 1:5 using a conductivity metre (DDS-11A, INESA, China)^35^, and the current in the EK tests was recorded periodically using an ammeter.

### *n*-Hexadecane analysis

The *n*-hexadecane was extracted using dichloromethane and acetone. First, a 3.0 g freeze-dried soil sample was mixed with 30 mL of dichloromethane and acetone (1:1, v/v) in a 250 mL conical flask, and then the following extraction steps were performed three times: (1) shaking for 30 min at 180 rpm, (2) immersion in an ultrasonic bath for 10 min, and (3) centrifugation for 2 min at a speed of 8000 rpm. A total of 90 mL of dichloromethane and acetone (1:1, v/v) were used for the whole extraction. The supernatant from the extraction steps was concentrated in a pear-shaped flask and then dissolved in 5.0 mL *n*-hexane for quantitative analysis. A Thermo Scientific TRACE gas chromatograph equipped with a flame ionization detector (GC-FID) was used for the analysis. An HP-5 capillary column was used with highly pure nitrogen as a carrier gas at a constant flow rate of 1.0 mL·min^−1^. The temperatures of the injector and detector were 250 °C and 300 °C, respectively. The temperature rose from 60 °C to 290 °C at 15 °C min^−1^ and was maintained at 290 °C for 5 min. The concentration of *n*-hexadecane was calculated using an external standard method for the authentic standard substance.

### DOM and water-soluble ion analyses

The DOM was extracted according to a method described by Kaiser *et al*.^36^, with some modifications. First, a 5.0 g freeze-dried soil sample was soaked in 25 mL distilled water. The suspension was shaken for 30 min and incubated at 20 °C overnight and then passed through 0.45 μm polysulfone membrane filters (Supor-450; Gelman Pall Sciences, Ann Arbor, MI). The concentration of total DOM in the filtrate, expressed as the dissolved organic carbon (DOC) concentration (Multi N/C 3000COC/TN), was determined. The total water-soluble ions, including K^+^, NH_4_^+^, NO_3_^−^, SO_4_^2−^ and PO_4_^3−^, that leached from the soil samples were measured according to Lu^35^, with some modifications. Freeze-dried soil (5.0 g) was immersed in 25 mL deionized water and maintained in suspension for 5 min. Then, it was left to settle, and the supernatant was filtered through a 0.45 μm Teflon filter, dried for 4 h at 105 °C, and weighed. During the drying process, 15% H_2_O_2 _was added to thoroughly remove the organic C.

### Dehydrogenase activity and microbial enumeration assay

The soil dehydrogenase (DHA) activity was measured by monitoring the rate of the reduction of triphenyl tetrazolium chloride (TTC) to triphenyl formazan (TPF), as described by Oliveira *et al*.^37^, with some modifications. TPF was detected using a spectrophotometer (UV-2550, Shimadzu) at 485 nm after a dark incubation for 24 h and expressed in μg TPF d·g^−1^ of dry soil 24 h^−1^.

The bacterial biomass was analysed using the real-time PCR of the 16S rRNA gene; this was performed with the ABI Prism 7000 Real-Time PCR Detection System (Applied Biosystems, USA) using SYBR *Premix Ex* Taq II (2×) and ROX Reference Dye (50×) (Takara, China). A standard curve was produced using genomic DNA extracted from *E. coli*. as a template to quantify the total number of bacterial 16S rRNA gene copies. The primers used for amplification of the 16S rRNA genes were 8F (5′-GAGAGTTTGATCCTGGCTCAG-3′) and 518R (5′-ATTACCGCGGCTGCTGG-3′). The conditions for the real-time PCR were 30 s at 95 °C and then 40 cycles of 95 °C for 15 s, 55 °C for 30 s, 72 °C for 45 s and 72 °C for 5 min.

### Statistical analysis

All measurements were carried out a total of four times (two times per treatment), and their means and standard deviations were calculated and plotted using SigmaPlot 10.0 (Systat Software, USA). SPSS 21.0 (USA) was used to analyse the variance and perform Pearson’s rank correlation analyses.

An estimate of the expected *n*-hexadecane residues for the Bio1-EK treatment was calculated as the sum of the residues in the Bio1 and EK only treatments,


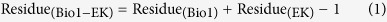


where Residue_(Bio1-EK)_ is the estimated *n*-hexadecane residue in the Bio1-EK treatment and Residue_(Bio1)_ and Residue_(EK)_ are the real *n*-hexadecane residues in the Bio1 and EK only treatments, respectively.

The amount of glucose to add to the Bio1 + EK1 + Bio2 + EK2 treatment (before “Bio2”) was defined according to the equation


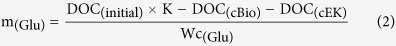


where m_(Glu)_ is the amount of glucose needed; DOC_(initial)_ is the original quantity of DOC in the uncontaminated soil; DOC_(cBio)_ and DOC_(cEK)_ are the residual content of DOC after 9 and 12 days of the cBio and cEK tests, respectively; Wc_(Glu)_ is the carbon content ratio of glucose, which is 40%; and K is the organic carbon ratio, accounting for the original amount according to the experimental design.

## Results

### Analysis of bioremediation, electrokinetic and bio-electrokinetic remediation processes

#### *n*-Hexadecane degradation

**Figure 2:**
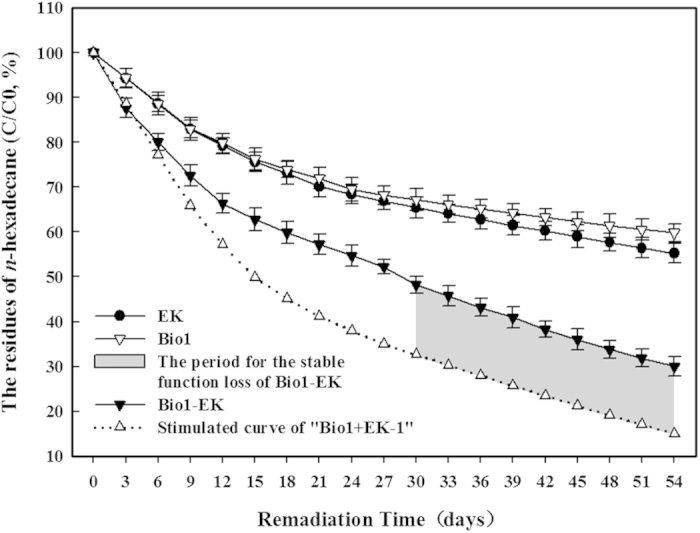
Residues of *n*-hexadecane in the EK, Bio1 and Bio1-EK treatments during the 54-day experimental period. Data shown are the means ± S.D. (n = 4).

According to the calculation of the residues shown in Fig. 2, the degradation extents of *n*-hexadecane in the Bio1 and EK treatments were 40.2% ± 2.0% and 44.8% ± 2.2%, respectively, after 54 days of remediation. Linear degradation rates occurred in both treatments during the first 9 days. Both treatments were relatively efficient at degrading *n*-hexadecane until 27 days had passed; after this point, the removal efficiencies remained low. The degradation rates in the Bio1-EK treatment were improved compared with those of the Bio1 (p < 0.05, 54 days) and EK (p < 0.05, 54 days) only treatments. Obviously higher rates with both treatment technologies working together in the Bio1-EK treatment occurred during the first 12 days. Then, the degradation rate of *n*-hexadecane started to decrease. In total, 69.9% ± 2.2% of the compound was removed by the end of the experiment (Fig. 2).

The curve of the *n*-hexadecane residue in the Bio1-EK treatment agreed well with the simulation (calculated by Equation (1)) during the initial phase. However, with further incubation, the degradation extent in the Bio1-EK treatment was prominently lower than expected, and there was a period where the degradation ratio of the Bio1-EK treatment was consistently 15.1% lower than expected (p < 0.05) (Fig. 2).

#### Soil DOC and appropriate content

**Figure 3:**
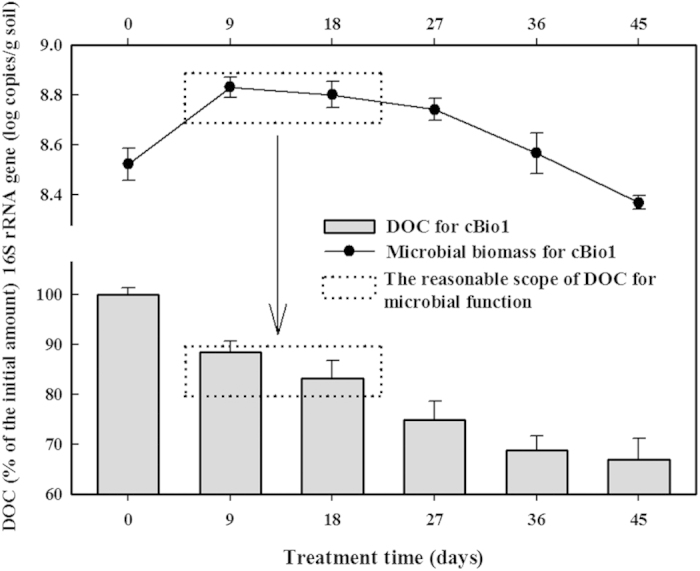
Total bacterial 16S rRNA gene copy number and DOC content (% of initial) in the cBio1 treatment during the 45-day experimental period. Data shown are the means ± S.D. (n = 4).

The amount of DOC decreased to 66.9% ± 1.7% of the original in the cBio treatment, a decline of 33.1% ± 2.1%, after 45 days (Fig. 3); this reflects on the importance of DOC as an easily available carbon source for soil microbial metabolism. During the first 9 days, 11.5% ± 1.5% of the DOC was consumed. The microbial biomass, estimated from the 16S rRNA gene copies, increased from the outset, corresponding to intensive DOC consumption. However, the DOC could compete with *n*-hexadecane as a carbon source for the microorganisms, which could have reduced the effectiveness of the bioremediation in the contaminated soil. There was a prominent decline in microbial biomass after 27 days of remediation, and after this point, the DOC content remained at 74.9% ± 3.8%, revealing that the DOC content was too low for the microorganisms. According to Fig. 3, the maintenance of the DOC content between 80% and 90% of the initial amount (marked by dotted lines in Fig. 3) should be an appropriate scope to minimize the competitive effects and extend the highly efficient period of bioremediation.

The bacterial biomass monitored in the Bio1 test reached 2.6 × 10^8^ copies·g^−1^ soil after 42 days, compared with 4.5 × 10^8^ copies·g^−1^ soil initially; that is, it was 42.5% lower after 42 days (p < 0.05), corresponding to the weak biodegradation of *n*-hexadecane shown in Fig. 2.

### Current, electrical conductivity and water-soluble ions during the electrokinetic remediation process

**Figure 4:**
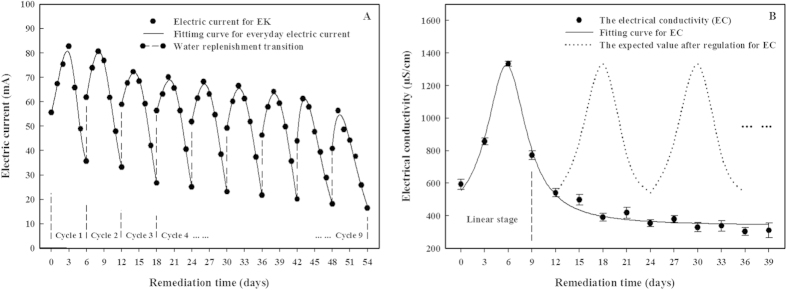
Variation of the electric current (**A**) and soil electrical conductivity (**B**) over time during the EK treatment. Data shown are the means ± S.D. (n = 4).

In the EK treatment, the electric current increased after water replenishment (which was done every 6 days), reached a peak and then decreased (Fig. 4A) until the water was replenished again. The maximum current was recorded during the first cycle, where it reached 82.8 mA after the third day. The increase after each water replenishment was less than that in the last cycle, and it eventually dropped to 56.3 mA. The re-injection of deionized water was periodically done to maintain a moisture content of 20% (w:w) and increase the current, which clearly happened; these results demonstrate that water replenishment is necessary to maintain a reasonable current during the EK treatments.

The variation of the soil EC in the composite samples (Fig. 4B) was induced by the applied electric field during the EK treatment. There was an obvious initial increase in EC, up to 1333 μs·cm^−1^ after 6 days. Then, the EC value rapidly decreased and was held at ca. 300–350 μs·cm^−1^, that is, it decreased by 73.7–77.5% (compared to its peak) after 39 days of remediation (p < 0.05). Although the water was replenished, the low maximum currents, arising from the low soil EC levels, also indicate that the effects of the electrodynamics and electrochemistry decreased after 9 days of treatment; this corresponds to the nonlinear decreases in the *n*-hexadecane residues during the EK treatment (Fig. 2). To restore the original electric current and sustain high soil EC levels, an ionic solution (like the nutrient solution used in this study) should be added during treatment; if this was done, the expected EC values would be higher (Fig. 4B).

**Figure 5:**
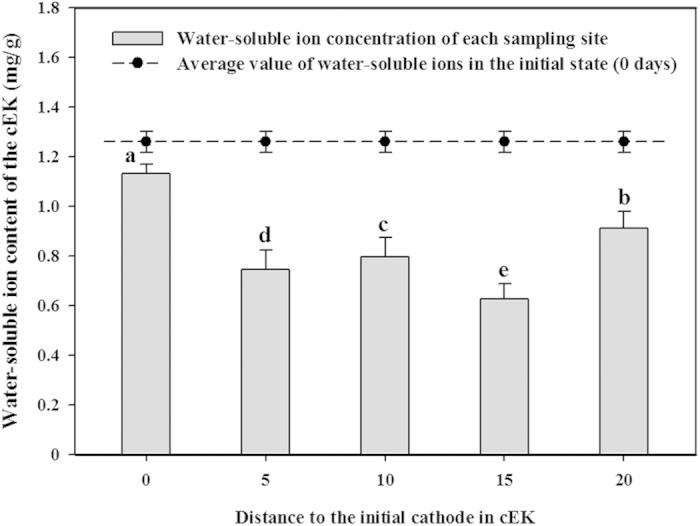
Variations in the distribution (column) and overall average amount (scatter) of the water-soluble ions in the cEK treatment after 9 days. Different small letters above the columns indicate significant differences among the samples (p < 0.05). Data are shown as the means ± S.D. (n = 4).

There was an obvious non-uniform distribution of the water-soluble ions in the cEK treatment, with prominent aggregations located in the vicinity of both electrodes after 9 days of treatment and the completion of a whole polarity cycle (Fig. 5); there was an average of 48.5% ± 1.7% more water-soluble ions at the electrodes than in the middle regions of the electric field (p < 0.05). Furthermore, there were more ions concentrated around the initial cathode compared with the initial anode (p < 0.05). Remarkably, the average quantity of the water-soluble ions was 32.9% ± 6.3% less in 9 days treatment than in the initial sample (0 days) (scatter; Fig. 5) (p < 0.01).

#### Effect of electrokinetics on bioremediation

**Figure 6:**
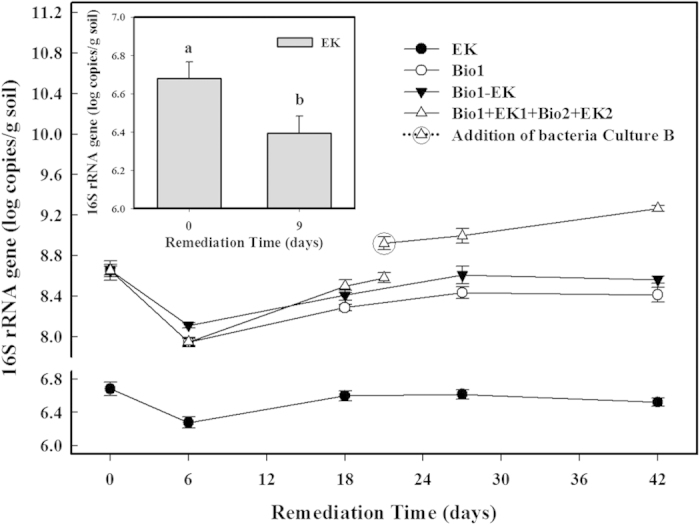
Total bacterial 16S rRNA gene copy numbers and a comparison of the total bacterial amounts at days 0 and 9 days during the EK-only treatment (p < 0.05) (Insert). Data are shown as the means ± S.D. (n = 4).

Even though the *n*-hexadecane degradation appeared to be improved in the Bio1-EK treatment compared with the Bio1 and EK treatments, the electrokinetics detrimentally affected the microorganisms in the later phases of the Bio1-EK treatment. This was demonstrated indirectly in the consumption of the DOC (the DOC content in the cEK treatment was 31.7 ± 1.4 mg·kg^−1^ after 9 days of treatment, a decrease of 16.2% ± 3.7% of the natural DOC content in the soil sample (p < 0.05) (Fig. 3)) and directly in the microbial numbers and DHA levels. The number of 16S rRNA copies after 9 days of the EK treatment was just 10^6^ copies·g^−1^ soil because there was no addition of exogenous oil-degrading bacteria; this was 48.2% ± 2.4% less than in the natural soil sample at 0 days (p < 0.05) (Fig. 6). Similarly, a decrease of 18.8% ± 1.8% in the bacterial number was achieved after 42 days of treatment in the Bio1-EK treatment (Fig. 6) (p < 0.05).

**Figure 7:**
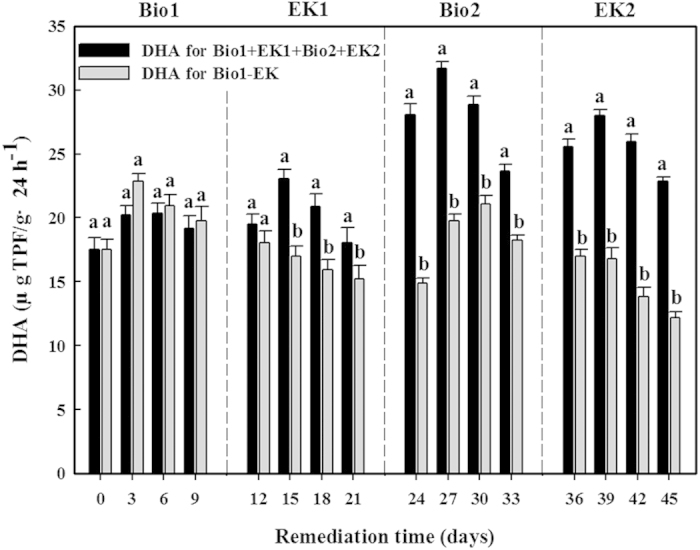
DHA for Bio1-EK and Bio1 + EK1 + Bio2 + EK2 tests. Different small letters above the columns indicate significant differences in DHA activity between two tests (p < 0.05). Error bars represent ± S.D. (n = 4).

The DHA activity of the soil samples in the Bio1-EK test decreased by 10.6 ± 0.4 μg TPF·g^−1^ dry soil 24 h^−1^ between 3 (maximum observed) and 42 (minimum observed) days of treatment (Fig. 7), and a highly significant positive correlation was observed between the degradation extent of *n*-hexadecane and the DHA activity (R = 0.587, p < 0.05, n = 16), even though part of the *n*-hexadecane was degraded by the electrokinetic treatment in Bio1-EK.

All of these factors indicate the nonlinear remediation rate over the Bio1-EK treatment duration (Fig. 2) and suggest that there was interference between the electrokinetic and bioremediation technologies during the long-term treatment when conducted simultaneously. Hence, alternating the two technologies (electrokinetics and bioremediation) might optimize each technology’s remediation efficiency and alleviate the interference between them, leading to a maximally efficient remediation process.

### Remediation efficiency using alternating electrokinetic and bioremediation technologies

#### Assessment of the alternating pattern

Because of the interference between the electrokinetic and bioremediation technologies, we investigated the application of bioremediation (Bio1) prior to the electrokinetics (EK1) and then prolonged the process by repeating the two treatment cycles (Bio1 + EK1 + Bio2 + EK2). During the treatment, the soil micro-environment was adjusted to optimize the remediation effects. In cBio1, 4.9 mg·kg^−1^ of DOC was consumed after 9 days of treatment, accounting for 11.5% ± 1.3% of the total DOC present (Fig. 3), and during the 12 days of the cEK treatment, 7.5 mg·kg^−1^ (19.9% ± 1.6% of the total DOC) was consumed. Therefore, the amount of DOC consumed through Bio1 + EK1 was estimated to be 12.4 mg·kg^−1^. To create the optimum DOC conditions (80–90% of original DOC; Fig. 3) for the second half of the Bio1 + EK1 + Bio2 + EK2 treatment (Bio2 + EK2), 20.5 mg·kg^−1^ glucose (calculated by Equation (2)) was mixed into the soil (to achieve 90% of original DOC content) before the Bio2 stage. The content of inorganic ions dissolved in the nutrient solution was the same as that used in the Bio1 bacterial culture (see ‘Bacteria cultures’). The soil was also supplemented with Culture B prior to Bio2 because of the decreases in the biomass and DHA activity of the bacteria in the cBio1 and Bio1 tests over time. The bacteria observed in the EK1 stage soil samples were enriched with Culture B, which were adapted to organic pollution, including the intermediate products of *n*-hexadecane degradation. Therefore, the microorganisms present favoured the acceleration of the bioremediation process.

#### *n*-Hexadecane degradation

**Figure 8:**
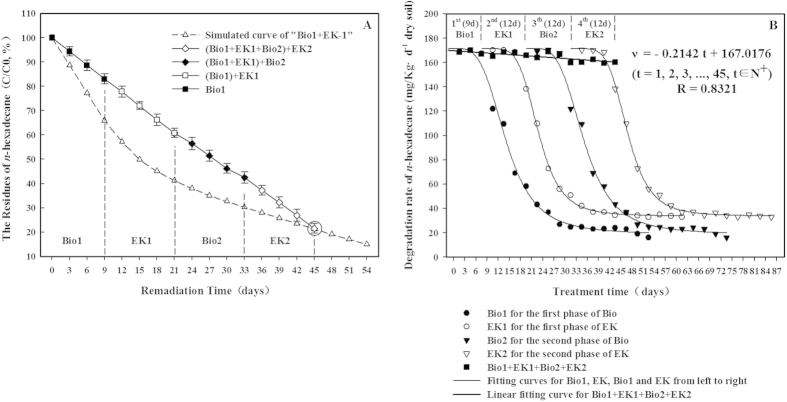
*n*-Hexadecane residual levels (**A**) and degradation rates (**B**) during the Bio1 + EK1 + Bio2 + EK2 treatment (alternating the bioremediation and electrokinetic technologies). Bio is the treatment applied with bacterial Culture A. Data shown are the means ± S.D (n = 4).

The residual levels of *n*-hexadecane in the Bio1 + EK1 + Bio2 + EK2 treatment, presented in Fig. 8A, show a linear decrease in the compound over time. After 39 days, 67.7% ± 2.2% of the *n*-hexadecane was removed, an improvement of 8.7% compared to the Bio1-EK test (p < 0.05), and 78.5% ± 2.0% of the *n*-hexadecane had been degraded after 45 days of treatment. The predicted removal of *n*-hexadecane, according to the combined effects of the Bio1 and EK treatments (simulated curve Bio1 + EK; Fig. 8A), was the same as the observed results of the Bio1 + EK1 + Bio2 + EK2 treatment after 45 days (circle; Fig. 8A).

The degradation rates of *n*-hexadecane during the Bio1 and EK treatments superimposed over that observed for the Bio1 + EK1 + Bio2 + EK2 treatment (Fig. 8B) show why the degradation rates during the Bio1 + EK1 + Bio2 + EK2 test remained high. The degradation rates for the Bio1 and EK treatments were consistently high during the initial 9–12 days and then declined. During the Bio1 + EK1 + Bio2 + EK2 treatment, the degradation rates remained linear throughout because the first part of the Bio1 or EK1 treatment curves were captured each time the treatment technology was alternated. Therefore, a near constant degradation rate of 167.0 mg·kg^−1^·d^−1^ *n*-hexadecane from the dry soil was achieved.

The estimated values of the degradation rates for each treatment process, based on the curves, were similar to the measured values listed in Table 3. It is interesting that the sum of the estimated values for the linear stages of each individual technology was almost equal to the estimated values of the Bio1 + EK1 + Bio2 + EK2 treatment, as was the observed degradation extent (Table 3). The results indicate that the EK stages in the Bio1 + EK1 + Bio2 + EK2 test (EK1 and EK2) had similar degradation efficiencies to those estimated from the Bio1-EK treatment upon extending the linear part of the curve to 12 days, compared to the original 9 days.

### Microbial biomass, DHA activity and soil electrical conductivity

**Figure 9:**
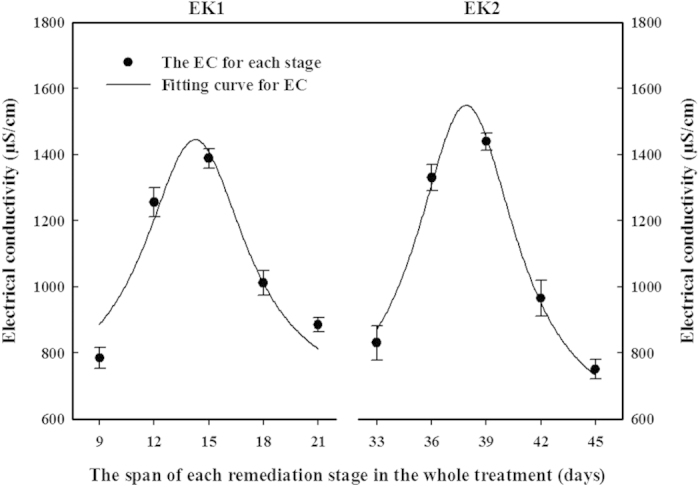
Soil electrical conductivity within the EK1 and EK2 stages of the Bio1 + EK1 + Bio2 + EK2 treatment. Data shown are the means ± S.D. (n = 4).

The variation of the soil EC during the electrokinetic stages of the EK1 + Bio1 + EK2 + Bio2 treatment shows that relatively high EC levels were maintained, as a result of the replenishment of the inorganic ions (Fig. 9). Compared with the soil EC during the EK treatment, which was relatively low after 9 days of treatment (Fig. 4B), the soils during the EK2 stage of the EK1 + Bio1 + EK2 + Bio2 treatment possessed EC values in the range of 751–1440 μs·cm^−1^. The EC met, and even exceeded, the value expected based on the inorganic ion supplement (dotted lines in Fig. 4B).

The bacterial biomass fluctuated during the Bio1 + EK1 + Bio2 + EK2 treatment (Fig. 6). A decrease in the copy number occurred from the outset of the Bio1 stage. The numbers started to increase after 6 days until the end of the experiment. The gene copy number prominently increased at 21 days, after the addition of Culture B, containing bacteria cultured from the final soil sample taken during the EK1 stage. The numbers then peaked at 10^9^ copies·g^−1^ soil after 42 days. The high microbe number ensured, to a certain extent, efficient microbial degradation during the long-term process.

The DHA activity of the soil samples in the Bio1 + EK1 + Bio2 + EK2 treatment remained high compared to that in the Bio1-EK treatment, especially during the EK1 stage and even more so during the Bio2 and EK2 stages (Fig. 7). The maximum DHA activity achieved was 31.7 ± 0.5 μg TPF·g^−1^ dry soil 24 h^−1^, and 22.5 ± 0.4 μg TPF·g^−1^ dry soil 24 h^−1^ of DHA activity was observed at the end of the test. Because of the DOC, inorganic ion and bacteria additions, the Bio1 + EK1 + Bio2 + EK2 treatment had a higher DHA activity than the Bio1-EK treatment (p < 0.05), except for during the Bio1 stage.

## Discussion

The linear degradation of *n*-hexadecane observed throughout the Bio1 + EK1 + Bio2 + EK2 treatment could clearly be attributed to the use of alternating bioremediation and electrokinetic technologies. The synergistic effect of bioremediation and electrokinetics during simultaneous application (e.g., Bio1-EK) has been demonstrated before^32^ and was observed here in the accelerated degradation of *n*-hexadecane during days 27–30 in the Bio1-EK treatment. However, the high remediation rates in the Bio1-EK treatment were not maintained. This was mainly because of the changes in the soil’s micro-environment^38^. During the bioremediation process, the easily utilizable carbon, such as DOM, was consumed from the soil micro-environment. Langwaldt *et al*.^39^ indicated that the labile part of the DOC, serving as a secondary carbon resource in the presence of chlorophenol contamination, helped to sustain the growth of bacteria. In another study, a marked increase in the soil microbial biomass was stimulated by the input of DOC substrates^40^. High organic matter (OM) content is typically associated with high microbial numbers^41^,^42^ and conversely, low levels of OM and low microbial numbers co-occur in subsurface soils^43^. These studies indicated that DOC functioned as an energy source that supported the microbial biomass in soils. With a similar conclusion, in this study, 11.5% ± 1.5% of the total DOC content was consumed, corresponding to the sharp rise in bacterial number during the first 9 days of testing. To extend the high bioremediation rates, the maintenance of the DOC, by adding an exogenous carbon source such as glucose, was necessary. Maintaining the organic carbon content at the natural levels in this study avoided the excessive excitation of microorganisms to utilize the exogenous carbon instead of the organic pollutant; we found that keeping 90% of the overall organic carbon in the soil allowed for *n*-hexadecane metabolism by the microorganisms, saved costs and maintained a near-original soil micro-environment.

Because of the interference of *n*-hexadecane in extracting DOC, the DOC consumption levels in the cBio1 and cEK treatments were used to estimate the quantity lost during the Bio1 and EK1 phases of the Bio1 + EK1 + Bio2 + EK2 treatment. The number of bacteria in the cBio treatment was higher than that in the Bio1 + EK1 stages of the Bio1 + EK1 + Bio2 + EK2 treatment after 18 days of testing, even though there was another carbon source (*n*-hexadecane) that could be metabolized in the soils of the latter treatment. This means that the sum of the DOC consumptions in cBio1 and cEK tests was likely more than that in the Bio1 + EK1 stage, and the quantity of DOC needed for the subsequent Bio2 + EK2 stages, as estimated from the cBio1 and cEK treatments, was likely appropriate. The higher microbial biomass and soil EC during the Bio2 + EK2 stages proved that the solution added before the Bio2 phase was appropriate to achieve the consistently high remediation efficiency observed during the Bio1 + EK1 + Bio2 + EK2 treatment.

Furthermore, the activity of the soil enzymes that are mainly responsible for pollutant degradation could be associated with the presence of DOM. Zhan *et al*.^44^ reported that DOM might counteract the inhibition on the soil enzyme activities induced by polycyclic aromatic hydrocarbons. The activity of dehydrogenase, a typical endoenzyme that can catalyse the dehydrogenation of organic compounds, correlated well with the soil microbial biomass (R = 0.9675)^45^, which was induced by the abundance of DOC. Thus, the *n*-hexadecane removal by bioremediation probably decreased with the consumption of DOM, implying the necessity of the organic carbon supplementation.

An analogous conclusion was drawn for the electrokinetic process. The primary mechanism of direct hydrophobic organic compound (HOCs) degradation was electrochemical oxidation, the effect of which was dependent on the strength of the electric current^46^,^47^,^48^. In the EK treatment, a significant positive correlation between the maximum electric current and the daily average removal efficiency of *n*-hexadecane was observed (R = 0.880, p < 0.01, n = 9). There have been similar reports on the removal of petroleum^29^, pyrene^49^ and cypermethrin pesticide^50^ by electrokinetic remediation technologies. The decline in the degradation rate with the decrease of the current occurred (due to the higher electrical resistance) because (1) the lower moisture content and lower availability of transferable ions led to weakened electromigration rates of the ions and (2) there was a lower soil EC. These changes attenuated the electrochemical oxidation efficiency. Some of the amendments implemented recovered the deficiencies and maintained a high oxidation ability in the Bio1 + EK1 + Bio2 + EK2 treatment. An obvious increase in the electric current occurred after water replenishment, which was attributed to the desorption of inorganic ions by electrochemical oxidation^24^ and the high concentrations of H^+^ and OH^−^, produced by the electrode reactions of water^51^. In a separate study, an increase in the electrolyte concentration, through the addition of a nutrient solution, resulted in a rise in the electrical conductivity and current, with the same conclusion^52^. In this study, the regulation of the water and inorganic ion contents helped to maintain the high degradation rates observed during the EK stages of the Bio1 + EK1 + Bio2 + EK2 treatment.

According to this study, the total water-soluble ion level in the soil was the crucial factor for both remediation technologies. Generally, polarity reversal has been shown to promote a relatively even distribution of ions^22^,^53^. However, in this study, the distribution of water-soluble ions was heterogeneous after a complete cycle (4 h) in a non-uniform field formed with columnar electrodes. The highest concentration of ions occurred around the electrodes, with the maximum field intensity, and the ion concentrations decreased with the decrease in the field intensity, positioned between the homopolar electrodes and in the centre of the field. In addition, smaller amounts of cations precipitated under alkaline conditions near the cathode compared to the number of anions around the anode. Similar results, showing that an acid pH favoured the migration of ions and hindered their precipitation (especially for metals), have been previously reported^24^,^54^. Furthermore, oxidation–reduction reactions would have occurred around the electrodes^19^. In addition to this, once the ions had precipitated or been adsorbed by the SOM, it would then take some time for the ions to dissolve again once the polarity was reversed and acidic conditions were created. This process produced a time lag that shortened the net migration duration and caused asymmetric migration, leading to the non-uniform distribution of ions. This was one of the underlying causes of the gradual weakening observed in the electric current over time and might compromise the sustainability and efficiency of the electrokinetic remediation technologies. The results prove that supplementing the soil with inorganic ions is essential to creating the ideal EK remediation conditions.

The order of the alternating Bio and EK treatments was restricted because of the impact of the two technologies on the soil properties, which were supposed to be favourable for the next remediation stage. As discussed above, the soil DOM not only affects bioremediation but also is a key factor to be considered in the alternating bioremediation and electrokinetic treatment. As the easily assimilated component of SOM, the DOM was the principal or underlying source of nutrients and energy with the maximum bioavailability to soil microorganisms^55^. Previous research^56^ has indicated that DOC can combine with hydrophobic organic compounds and thus make the bioavailability of contaminants increase in the aqueous phase. However, electrochemical oxidation did harm the DOM in an effective mass transfer system (using electroosmosis) and counteracted the benefits of the DOM. Some studies^57^ have observed an increase in the DOC, to some extent, after treatment with electrokinetics; however, the increase was achieved under acid soil conditions, as opposed to the alkaline soil in this experiment. Thus, employing electrokinetic treatment before bioremediation would have adversely affected the overall degradation efficiency of the alternating treatment.

For the microbial viability, the alternating pattern avoided the effects of electricity and *n*-hexadecane in combination on the microorganisms, providing them with more time to adapt to the extreme environment. Due to their source, from soil contaminated with petroleum, the microorganisms could maintain an ideal activity and viability while exposed to the pollutant for some time. Then, before the microbial metabolic activity fell, the use of a weak electric field caused both electrochemical oxidation and microbial stimulation. For the latter, there was an obvious increase in the cell density during the EK1 process, which was a little more than that observed in the Bio1-EK treatment after 18 days. The data indicated a promoting effect of the electrical current on microbial reproduction, in accordance with Li *et al*.^58^, and a stronger resistance due to adaptation to *n*-hexadecane and environmental stress. However, the indigenous bacteria were adversely affected and decreased during the initial phase, over 9 days, in the EK-only treatment (Fig. 6); that was another reason why the EK stage was applied after the Bio1 stage.

When the bacteria in Culture B were added for Bio2, the cell density increased sharply, and during EK2, the stimulatory effect from the electricity was again observed. It is noteworthy that the bacteria in Culture B were cultured from soil sampled at the end of EK1. Therefore, the *n*-hexadecane-degrading bacteria present in Bio2 had already acclimatized to the soil conditions but had been in a poor physiological state because of the environmental pressures. The intermediate products of *n*-alkanes have previously been shown to serve as a carbon and energy source for alkane-utilizing bacteria, creating an environment conducive to microbial survival^59^. This was the advantage of conducting the second bioremediation phase, as reflected in the quantity of bacteria during the Bio2 and EK2 stages.

Given the costs of the remediation technologies, alternating the technologies would reduce the energy consumption by half compared to applying the EK-only treatment for the same duration. Shortening the EK period also saves on electrode costs.

## Conclusion

The DOM content (mass ratio of 80–90% of original) is critical to optimize the bioremediation of alkane-contaminated soil, reducing the impacts of both competitive effects and nutritional deficiency. The water-soluble ions were positively correlated with the soil electrical conductivity. Supplementation maintained the EC value of 1300–1400 μs·cm^−1^ to stabilize the electrokinetic efficiency. Through the regulation of the DOC and water-soluble ion contents, the integration of bioremediation and electrokinetics combined four efficient remediation processes. The kinetic curves of *n*-hexadecane degradation approximate straight lines, and a uniform degradation rate of 167.0 mg·kg^−1^·d^−1^ of *n*-hexadecane was achieved throughout the whole process. The alternating technology ameliorated the adverse effects of the current electrokinetic-bioremediation treatment to maximize the extent and further improved the remediation efficiency of *n*-hexadecane-contaminated soil.

## Additional Information

**How to cite this article**: Wang, S. *et al*. Effect of alternating bioremediation and electrokinetics on the remediation of *n*-hexadecane-contaminated soil. *Sci. Rep*. **6**, 23833; doi: 10.1038/srep23833 (2016).

## Figures and Tables

**Table 1:** Initial characteristics of the soil used in the experiment (after being air-dried and sieved through a 2 mm mesh).

Soil properties	Value
pH	7.65
CEC (cmol·kg^−1^)	32.66
Organic C (OC) (g·kg^−1^)	10.50
DOC:OC	0.36
Soil Texture (mm, %)	
<0.002	12.3
0002–0.02	21.4
0.02–2.00	66.3

**Table 2:** Overview of the experimental treatments applied.

Experiment	*n*-hexadecane (mg·kg^−1^ dry soil)	Bacteria- Culture A (copies·g^−1^ dry soil)^a^	Bacteria- Culture B (copies·g^−1^ dry soil)^a^	Nutrient solution^b^	Inorganic ions^b^	Rehydration (once every 6 days)^b^	Applied voltage (V)	Polarity reversal time (h)
cBio1	0	4.5 × 10^8^	0	−	+	+	0	0
cEK	0	0	0	−	+	+	24	2
CK	8944	0	0	−	−	+	0	0
Bio1	8944	4.5 × 10^8^	0	+	−	+	0	0
EK	8944	0	0	+	−	+	24	2
Bio1-EK	8944	4.5 × 10^8^	0	+	−	+	24	2
Bio1 + EK1 + Bio2 + EK2	8944	4.5 × 10^8^	4.5 × 10^8^	+	−	+	24^c^	2^c^

^a^The values represent the final concentrations of bacteria after being mixed into the soil.

^b^“+” and “−” represent addition and no addition, respectively.

^c^The electric field was only applied during the electrokinetic remediation stages (EK1 and EK2).

**Table 3:** Contrast of *n*-hexadecane degradation among different treatments corresponding to the respective time sections.

Treatment	R^2^	Total amount degraded over 45 days (mg·Kg^−1^)		Estimates (based on fitted values) of the degradation levels of each stage (mg·Kg^−1^)				Total amount degraded over 45 days based on alternating the treatment technologies (mg·Kg^−1^)	
		Fitted value^a^	Measured value^c^	1^st^ (9d) Bio1	2^nd^ (12d) EK1	3^th^ (12d) Bio2	4^th^ (12d) EK2	Fitted value	Measured value
Bio	0.9940	3565.85 (39.9%)^b^	3399.04 (38.0%)	1496.68^d^ (16.7%)	–	1931.77^d^ (21.6%)	–	7280.54^e^ (81.4%)	7017.42 (78.5%)
EK	0.9984	3857.31 (43.1%)	3702.80 (41.4%)	–	1926.04^d^ (21.5%)	–	1926.04^d^ (21.5%)		
Bio1 + EK + Bio2 + EK2	0.6923	7282.53^g^ (81.5%)	7017.42 (78.5%)	1493.83^f^ (17.0%)	1962.74^f^ (22.0%)	1929.57^f^ (21.4%)	1896.39^f^ (21.0%)	7282.53^g^ (81.5%)	

^a^Fitted values represent the area below the curves, determined using Sigmaplot V10.0. The equation of the curve for Bio1 + EK1 + Bio2 + EK2 was *v*_(*Bio*1+*EK*1+*Bio*2+*EK*2)_ = −0.2142t + 167.0176.

^b^The percentage degraded of the initial content is shown in brackets.

^c^The measured values were determined by gas chromatography.

^d^Fitted values of the different “bioremediation” and “electrokinetics” stages during the Bio1 + EK1 + Bio2 + EK2 treatment were determined using Sigmaplot V10.0, in terms of the treatment time, respectively.

^e^The sum of the fitted values for each stage (d).

^f^The fitted values of different stages from “Bio1 + EK1 + Bio2 + EK2”, determined by Sigmaplot V10.0 according to the treatment time of each stage.

^g^Total amount of the fitted values shown for each stage (f).
